# Severe Hyponatraemia Following Underwater Colonoscopy and Polypectomy in an Elderly Woman: A Multifactorial Case Involving Bowel Prep, Procedural Stress, and Occult Malignancy

**DOI:** 10.7759/cureus.90810

**Published:** 2025-08-23

**Authors:** Shashank Ramakrishnan, Priya Goswami, Freya Dow, Sahin Ocakkuran, Dariush Sadigh

**Affiliations:** 1 Gastroenterology, Whittington Health NHS Trust, London, GBR; 2 General Practice, Royal Free London NHS Trust, London, GBR

**Keywords:** bowel cleansing agents, colonoscopy complications, colorectal cancer, elderly people, gastroenterology and endoscopy, hypertonic sodium chloride, hyponatraemia, paraneoplastic syndromes, syndrome of inappropriate secretion of antidiuretic hormone (siadh), underwater polypectomy

## Abstract

We report a case of a 77-year-old woman with a previously normal serum sodium of 141 mmol/L one week earlier, who developed acute severe symptomatic hyponatraemia (serum sodium 106 mmol/L) two days after an uneventful underwater colonoscopy and polypectomy for a caecal lateral spreading tumour (LST-G). The procedure was preceded by a positive faecal immunochemical test (FIT) in April 2025, followed by a CT colonogram showing a caecal lesion. She completed a low-solute Plenvu® (Norgine, Amsterdam, Netherlands) bowel preparation prior to endoscopy. Collateral history revealed three falls and two episodes of vomiting in the 24 hours before hospital presentation. On admission, she was found confused, with laboratory features consistent with syndrome of inappropriate antidiuretic hormone secretion (SIADH), including a urine sodium of 83 mmol/L. She received 3% hypertonic saline in intensive care, with full neurological recovery. Histology confirmed a T2N0M0 adenocarcinoma. This case highlights the importance of recognising risk factors for hyponatraemia - including procedural stress, low-solute preparation, age, and occult malignancy - particularly in the setting of water-assisted colonoscopy, and supports early biochemical monitoring in high-risk patients.

## Introduction

Colonoscopy is routinely performed and generally safe. Hyponatraemia is a recognised but uncommon complication, usually attributed to excessive free water intake and low-solute diet during bowel preparation. A 2003 prospective study reported hyponatraemia in ~11% of patients post-preparation, though mostly mild and asymptomatic [1]. More recent systematic reviews report pooled rates of hyponatraemia around 0.9% with sodium-phosphate preparations and 3.3% with polyethylene glycol (PEG)-based regimens [2]. 

Severe symptomatic hyponatraemia, however, is exceptionally rare. Multiple mechanisms may contribute, including excessive hypotonic fluid intake, impaired renal free water excretion due to low dietary solute, and non-osmotic anti-diuretic hormone (ADH) release triggered by nausea, vomiting, or procedural stress [3]. Elderly patients are especially vulnerable due to physiological decline in renal concentrating ability [4]. In addition, syndrome of inappropriate antidiuretic hormone secretion (SIADH) can be precipitated by malignancy, vomiting, or procedural stress. 

Water-assisted colonoscopy refers to the use of water rather than air or CO₂ for colonic insufflation during insertion. This technique improves mucosal visualisation and patient comfort [5]. In underwater endoscopic mucosal resection (UEMR), water immersion also flattens folds and aids polyp resection. Although UEMR is generally safe, isolated case reports have linked it to water intoxication and symptomatic hyponatraemia, potentially via transmucosal water absorption [6,7]. The colon is the body’s primary site of water reabsorption, and direct exposure to hypotonic irrigation fluid may therefore allow systemic uptake. This mechanism is analogous to transurethral resection of prostate (TURP) syndrome [8]. 

We present a rare case of severe hyponatraemia following water-assisted colonoscopy involving multiple risk factors. 

## Case presentation

A 77-year-old woman with hypertension, atrial fibrillation (on apixaban), and a dual chamber pacemaker (implanted in 2007 for Mobitz type 2) was referred following a positive faecal immunochemical test (FIT) in April 2025 and CT colonography showing a caecal lesion. She had diverticular disease but no prior gastrointestinal malignancy. 

Her medications included ramipril 1.25 mg daily, apixaban 5 mg twice daily, bisoprolol 1.25 mg daily, and atorvastatin 20 mg nightly. 

She underwent a water-assisted colonoscopy and polypectomy on 19/05/2025 for a 3-cm granular-type lateral spreading tumour (LST-G) in the caecum. She completed a standard split-dose regimen of Plenvu® (Norgine, Amsterdam, Netherlands), taking two 500 mL doses approximately 12 and 4 hours before the procedure. She followed a low-residue diet and was instructed to avoid solid food the day before the procedure. Approximately 500 mL of water was instilled during the procedure, with most suctioned during withdrawal. While there are no formal studies prescribing an optimal volume of water for underwater colonoscopy, this is broadly similar to amounts described in technical reports of underwater endoscopic resections, which typically report instillation in the range of 200-400 mL [5]. The procedure was otherwise uneventful. 

On 22/05/2025, she contacted the endoscopy team complaining of lethargy and dizziness. Neurological examination was normal, and outpatient blood tests were arranged, which later revealed a serum sodium of 112 mmol/L. Her most recent blood test, a week prior to the procedure (12/05/2025), showed a normal serum sodium of 141 mmol/L. She could not be contacted, prompting a welfare check. 

On 23/05/2025, she was found confused at home and brought to the emergency department. Collateral history revealed that she had experienced three falls and two episodes of vomiting in the 24 hours prior to presentation. A venous blood gas showed sodium of 101 mmol/L; formal labs confirmed sodium of 106 mmol/L. Laboratory investigations on admission demonstrated biochemical features consistent with SIADH, including a low serum osmolality and inappropriately concentrated urine (Table 1).

**Table 1:** Lab results on presentation to hospital on 23/05/2025 Abbreviations: TSH, thyroid-stimulating hormone; T3, triiodothyronine; T4, thyroxine.

Laboratory investigations 23/05/2025 08.30	Report	Reference range
Sodium	106 mmol/L	(135-145 mmol/L)
Urine sodium	83 mmol/L	
Serum osmolality	219 mOsmol/kg	(275-295 mOsmol/kg)
Urine osmolality	272 mOsmol/kg	
Potassium	3.6 mmol/L	(3.5 - 5.3 mmol/L)
Urea	1.8 mmol/L	(2.5 - 7.8 mmol/L)
Creatinine	28 μmol/L	(45-85 μmol/L)
Adjusted Calcium	2.19 mmol/L	(2.2 – 2.6 mmol/L)
Phosphate	0.53 mmol/L	(0.8 1.5 mmol/L)
TSH	0.44 mIU/L	(0.27 - 4.2 mIU/L)
Free T3	1.9 pmol/L	(3.10 – 6.80 pmol/L)
Free T4	17.4 pmol/L	(11.9 – 21.6 pmol/L)
Cortisol	1269 nmol/l	(133-537 nmol/L)

She was 72 kg and 1.65 m (BMI 26.5 kg/m^2^). Estimated total body water (0.45-0.50 × weight) was ~32-36 L. With baseline sodium 141 mmol/L and admission sodium 106 mmol/L, the sodium deficit was ~1,200 mmol. 

**Figure 1:**
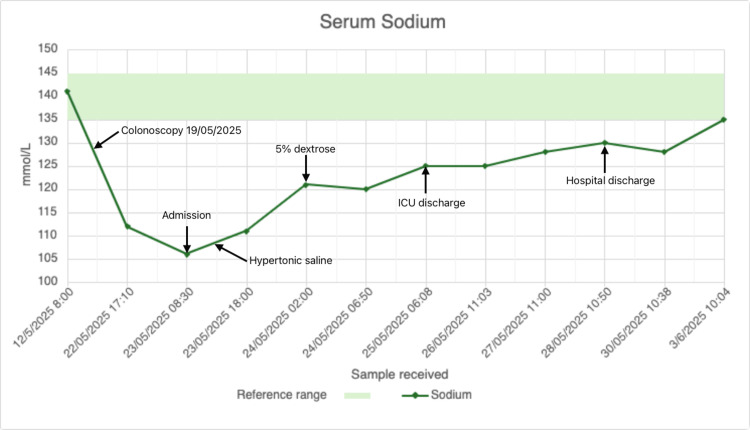
Serum sodium trend during admission and follow-up. Serum sodium fell from a baseline of 141 mmol/L (12/05/2025) to 106 mmol/L on admission (23/05/2025), four days after water-assisted colonoscopy and polypectomy. Following initial isotonic saline, hypertonic saline was administered in the intensive therapy unit (ITU), resulting in a rise from 106 to 120 mmol/L within 22 hours. An interim blood gas sample at 02:00 on 24/05/2025 showed sodium of 121 mmol/L, prompting 5% dextrose to prevent further correction. Subsequent serum sodium stabilised, with recovery supported by spontaneous aquaresis. The patient was discharged on 28/05/2025 with sodium at 130 mmol/L and maintained normal sodium on follow-up. Reference range: 135–145 mmol/L (green shaded area).

Initial management included 1 L of intravenous 0.9% saline, given for presumed dehydration following bowel preparation. She was then transferred to the intensive therapy unit (ITU), where she received 500 mL 3% hypertonic saline. Serum sodium rose from 106 mmol/L (08:30, 23/05) to 120 mmol/L (06:50, 24/05), an increase of 14 mmol/L in ~22 hours. An interim blood gas sample at 02:00 on 24/05 showed sodium of 121 mmol/L, prompting 5% dextrose to halt further rise. Her sodium stabilised at 120 mmol/L following this, with full neurological recovery. The patient’s sodium trend during admission and subsequent follow-up, including the response to hypertonic saline, is illustrated in Figure 1. 

Available fluid balance records are summarised in Table 2. Documentation was incomplete due to paper-based charts and the patient being transferred between the emergency department and intensive care on 23/05. From 23/05, she was managed with a 1.5L fluid restriction, which was lifted on 27/05 as her sodium had stabilised. From 24/05 onwards, recorded urine outputs exceeded intake, consistent with a spontaneous free water diuresis (aquaresis) as non-osmotic ADH secretion abated.

**Table 2:** Documented fluid balance during admission

Date	Total Input (L)	Total Output (L)	Net Balance
23/05	Not fully recorded	Not fully recorded	+283 mL
24/05	1.4	3.1	–1.7 L
25/05	1.5	2.8	–1.3 L
26/05	1.5	4.6	–3.1 L
27/05	Not recorded	Not recorded	Fluid restriction lifted
28/05	–	–	Discharged

She was discharged on 28/05/2025 with a serum sodium of 130 mmol/L. Histology confirmed T2N0M0 caecal adenocarcinoma. She declined surgery and opted for conservative management. 

## Discussion

**Figure 2:**
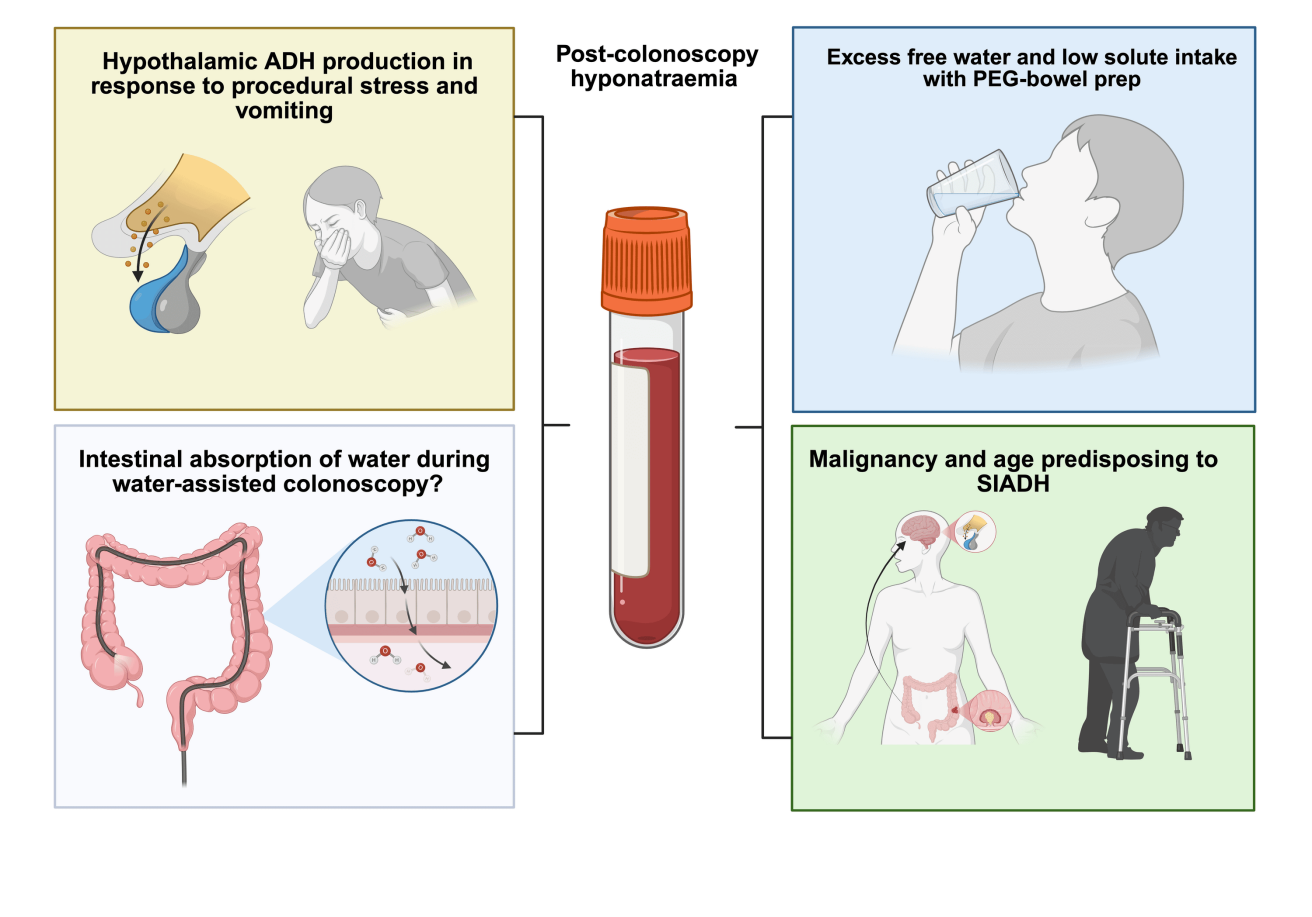
Suggested mechanisms contributing to acute hyponatraemia in this patient Procedural stress and vomiting likely stimulated hypothalamic ADH release, providing a potent non-osmotic trigger. The low-solute PEG bowel preparation, supplemented with ascorbic acid, reduced renal osmotic load and promoted intraluminal fluid retention, limiting free water excretion. Water immersion during colonoscopy may have permitted transmucosal absorption of hypotonic irrigation fluid, analogous to TURP syndrome. Finally, advanced age and the presence of a caecal adenocarcinoma predisposed to impaired water clearance and possible paraneoplastic SIADH, compounding the risk. Source: "Suggested mechanisms of hyponatraemia in our patient" by Ramakrishnan S, 11 August 2025. Created in BioRender. https://BioRender.com/4vbbdf0 Abbreviations: ADH, anti-diuretic hormone; PEG, polyethylene glycol; SIADH, syndrome of inappropriate antidiuretic hormone secretion; TURP, transurethral resection of prostate

This case illustrates a rare but clinically significant complication, acute symptomatic hyponatraemia after underwater colonoscopy, mediated by multiple factors (Figure 2).

At presentation, serum osmolality was 219 mOsm/kg (low), urine osmolality 272 mOsm/kg (inappropriately concentrated), and urine sodium 83 mmol/L - fulfilling SIADH diagnostic criteria in the context of euvolaemia and normal thyroid/adrenal function. 

Vomiting and procedural stress are potent non-osmotic stimuli for ADH release [3]. Elderly patients also have impaired renal free water clearance [4]. PEG-based bowel preparations, particularly in the setting of low dietary solute, predispose to water retention [2,4]. Adequate solute intake, particularly sodium and protein, is essential for renal free water clearance; ensuring high-risk patients resume a normal or supplemented diet promptly after bowel preparation may reduce this risk. 

Although colonic adenocarcinoma is not classically associated with SIADH, malignancy-related ADH dysregulation has been described. Caecal cancers are relatively uncommon, but there are case reports of SIADH in colorectal carcinoma, supporting its plausibility as a paraneoplastic phenomenon [9]. 

Plenvu® contains PEG combined with ascorbic acid, which enhances osmotic fluid retention in the bowel to improve cleansing efficacy [10,11]. While generally safe, this mechanism may contribute to electrolyte disturbances in susceptible patients, particularly where solute intake is low or free water intake is excessive. 

Given that one of the colon’s primary physiological functions is to absorb water, direct intraluminal exposure to hypotonic fluid during underwater endoscopy could, under certain conditions, lead to transmucosal free water uptake and dilutional hyponatraemia. In practice, only modest volumes are used (~200-500 mL, most suctioned), making this an unlikely sole cause, but partial absorption could contribute synergistically with other risk factors. This mechanism is supported by isolated case reports [6,7] and is analogous to TURP syndrome, where systemic irrigation fluid absorption is well recognised [8]. Prospective studies quantifying water absorption during underwater colonoscopy would help clarify this risk. 

Fluid balance records (Table 2) demonstrated urine outputs greater than intake after hypertonic therapy, consistent with a spontaneous free water diuresis (aquaresis). The patient’s sodium rose by 14 mmol/L within 22 hours of initiating hypertonic saline, exceeding the conventional 10-12 mmol/L/24 h safety threshold. Early recognition allowed correction with 5% dextrose, preventing further rise. This underscores the importance of close biochemical monitoring to avoid overcorrection, particularly as spontaneous aquaresis develops once non-osmotic ADH secretion abates.

This case also illustrates the potential for post-colonoscopy hyponatraemia to be misattributed to dehydration from bowel preparation. In such settings, isotonic saline alone is unlikely to correct sodium, and prompt recognition of SIADH physiology is essential to avoid delays in appropriate management. 

## Conclusions

Severe hyponatraemia is a rare but potentially life-threatening complication of colonoscopy. Elderly patients undergoing low-solute bowel preparation - particularly with water-assisted techniques - are at increased risk when multiple triggers coexist. Clinicians should maintain a high index of suspicion for SIADH in such contexts. 

Prevention strategies include pre- and post-procedural biochemical monitoring, patient education on fluid intake, electrolyte-guided regimens, and ensuring adequate solute (sodium and protein) intake in at-risk individuals. Future research should also explore whether dietary strategies or electrolyte-enriched supplements can reduce risk, and whether prospective studies can quantify intraluminal water absorption during underwater colonoscopy to guide monitoring protocols. 
